# Water Consumption Characteristics and Water Use Efficiency of Winter Wheat under Long-Term Nitrogen Fertilization Regimes in Northwest China

**DOI:** 10.1371/journal.pone.0098850

**Published:** 2014-06-06

**Authors:** Yangquanwei Zhong, Zhouping Shangguan

**Affiliations:** State Key Laboratory of Soil Erosion and Dryland Farming on the Loess Plateau, Northwest A & F University, Yangling, Shaanxi, P.R. China; Institute for Plant Protection (IPP), CNR, Italy

## Abstract

Water shortage and nitrogen (N) deficiency are the key factors limiting agricultural production in arid and semi-arid regions, and increasing agricultural productivity under rain-fed conditions often requires N management strategies. A field experiment on winter wheat (*Triticum aestivum* L.) was begun in 2004 to investigate effects of long-term N fertilization in the traditional pattern used for wheat in China. Using data collected over three consecutive years, commencing five years after the experiment began, the effects of N fertilization on wheat yield, evapotranspiration (ET) and water use efficiency (WUE, i.e. the ratio of grain yield to total ET in the crop growing season) were examined. In 2010, 2011 and 2012, N increased the yield of wheat cultivar Zhengmai No. 9023 by up to 61.1, 117.9 and 34.7%, respectively, and correspondingly in cultivar Changhan No. 58 by 58.4, 100.8 and 51.7%. N-applied treatments increased water consumption in different layers of 0–200 cm of soil and thus ET was significantly higher in N-applied than in non-N treatments. WUE was in the range of 1.0–2.09 kg/m^3^ for 2010, 2011 and 2012. N fertilization significantly increased WUE in 2010 and 2011, but not in 2012. The results indicated the following: (1) in this dryland farming system, increased N fertilization could raise wheat yield, and the drought-tolerant Changhan No. 58 showed a yield advantage in drought environments with high N fertilizer rates; (2) N application affected water consumption in different soil layers, and promoted wheat absorbing deeper soil water and so increased utilization of soil water; and (3) comprehensive consideration of yield and WUE of wheat indicated that the N rate of 270 kg/ha for Changhan No. 58 was better to avoid the risk of reduced production reduction due to lack of precipitation; however, under conditions of better soil moisture, the N rate of 180 kg/ha was more economic.

## Introduction

Northwest China is a vast semi-arid area with average annual precipitation in the range of 300–600 mm and more than 90% of the land is cropland [1]. This means that water is the primary factor limiting crop yields. In addition, world food demand is expected to double during 2005–2050 [2], thus it is important to increase food production with lower water use [3], particularly in water shortage regions. Currently, water stress and nutrient deficits are the main factors limiting primary production in arid and semi-arid environments [4]–[8]. Therefore, many rain-fed farming experts have focused on how to increase crop water use efficiency (WUE, i.e. the ratio of grain yield to total ET in the crop growing season) by irrigation and fertilization.

In the 1990 s, many studies on effects of limited irrigation on crop yields and WUE showed that by reducing irrigation volume, crop yield could be generally maintained and product quality improved [9]–[13],and appropriate irrigation management can increase crop yield and WUE [14]–[16]. There are several sources of soil water in irrigated or high water-table areas, however, precipitation is the only source of soil water for crop growth in many rain-fed farming systems of arid and semi-arid regions. Therefore, new methods need to be devised to improve WUE in this non-irrigated farming system.

N fertilization is a common practice to increase grain production, but its performance depends on soil water status [17]–[19]. The importance of increasing crop yield and improving soil quality through fertilization has been confirmed. The increasing use of N fertilizer could significantly increase maize production [8], [20], and already affects a large proportion of the world's food production [21], [22]. Fan et al. [1] reported that inorganic N and phosphorus (P) fertilization increased grain yields by 50–60% in China, and reports from Europe showed that N fertilizers can increase crop yield significantly [23]. N fertilization is well known to improve soil fertility [24], [25]; however, using excessive N fertilizer can decrease the N utilization rate, which not only causes a huge waste of resources and economic losses, but can also adversely impact the environment [26]–[28]. Balancing the N rate, WUE and yield is an important problem in dryland farming systems. Better understanding of interactions among precipitation, fertilization and crops production is essential for efficient utilizations of water resources and N fertilizers, and sustainable food productions in rain-fed cropping systems experiencing climate change [1]. Long-term fertilization experiments are valuable to follow crop yield, soil fertility, WUE and risk management over time [29], [30]. Various long-term experiments have examined how to increase yield and WUE of wheat, using irrigation, organic or inorganic fertilizer, soil tillage and crop management [31]–[33]. However, few experiments have been done on evapotranspiration (ET) and WUE under circumstances with only N fertilizer and without irrigation in northwest China. With China's urbanization, increasing numbers of farmers have abandoned farms to urban construction and this has led to a loss of labor. Thus, most farmland in northwest China region still uses traditional cropping practices that all fertilizer applied once prior to planting [1], lack of careful management of irrigation and other tasks. Kang et al. [14] reported that difference in yield and WUE are also related to regional variability in environment and crop varieties, so information specific to a region is needed for developing and refining the agricultural performance in this region. In these circumstances, it is very important to determine the advantages and disadvantages of long-term N fertilization on yield of different varieties.

This study examined two different water-sensitive cultivars of winter wheat (*Triticum aestivum* L.) to investigate effects of N fertilizers on crop yield, ET and WUE, using the most common management of farmers in northwest China. The objectives were (1) to investigate impacts of traditional long-term N fertilization on yields of two different water-sensitive wheat cultivars; (2) to examine the effect of N fertilizers on total ET, and soil water consumption from different soil layers of the two cultivars; and (3) to establish relationships among crop yield, WUE and ET and determine optimum N fertilizer rates in northwest China. This study may compensate for some of the lack of long-term influence only N fertilizer on crop production, and the results should provide guidelines to farmers in the region on choosing appropriate cultivars and obtaining high yields with appropriate N application.

## Materials and Methods

### Experiment site and climatic conditions

The study commenced in October 2004 in an experiment field of the Institute of Soil and Water Conservation of the Northwest A & F University, Yangling, Shaanxi (34°17′56″N, 108°04′7″E). Located on the southern boundary of the Loess Plateau, the experiment site has a temperate and semi-humid climate with a mean annual temperature of 13°C and a mean annual precipitation of 632 mm, of which about 60% occurs during July–September.

### Experiment design

The study adopted a randomized block design with three replications. Two winter wheat (*Triticum. aestivum* L.) cultivars were used: Zhengmai No. 9023 (ZM) is water sensitive and poorly drought-tolerant and Changhan No. 58 (CH) is drought-tolerant and suitable for drought prone environments. The thousand-kernel weights of ZM and CH were 43.58 and 43.61 g, respectively. N treatments were applied at five rates: 0, 90, 180, 270 and 360 kg/ha (N0, N90, N180, N270 and N360, respectively). Plot size was 2 m×3 m with 20 rows (15-cm spaces) of wheat sown at 90 seeds/row. Wheat was sown in early October and harvested in early June the following year. The seeding rate was 130 kg/ha. Immediately before sowing, the fertilizer was evenly spread on the soil surface and then incorporated into the upper 15 cm soil by chiseling. N was applied as urea and P (75 kg P_2_O_5_/ha) as super phosphate. No potassium fertilizer was applied, and the site was ploughed to bury weeds before sowing.

### Measurements

In all treatments, the volumetric soil water content was measured every 10 cm for 0–100 cm of soil and every 20 cm for 100–300 cm with a neutron moisture meter (CNC100, Super Energy, Nuclear Technology Ltd., Beijing, China). The 3-m-long neutron gauge access tube was buried vertically in the center of each plot at the beginning of the study. Soil water was measured during the first week of every wheat growing month except January and February. If any precipitation occurred just before or during the measurement period, then measurements were postponed for several days until the soil moisture attained a normal degree. The yields and the thousand-kernel weights of wheat in all plots were measured at harvest time in early June. Since this paper aimed to test the cumulative effects of N fertilization, we chose data from three wheat growing years with different precipitation characteristics: 2009–2010, 2010–2011 and 2011–2012.

### Calculation and statistics

ET of winter wheat was calculated using the following equation [33]:
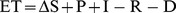



Where ΔS is soil water storage change, P is precipitation, I is irrigation rate, R is surface runoff and D is deep water percolation (all in mm).

**Figure 1:**
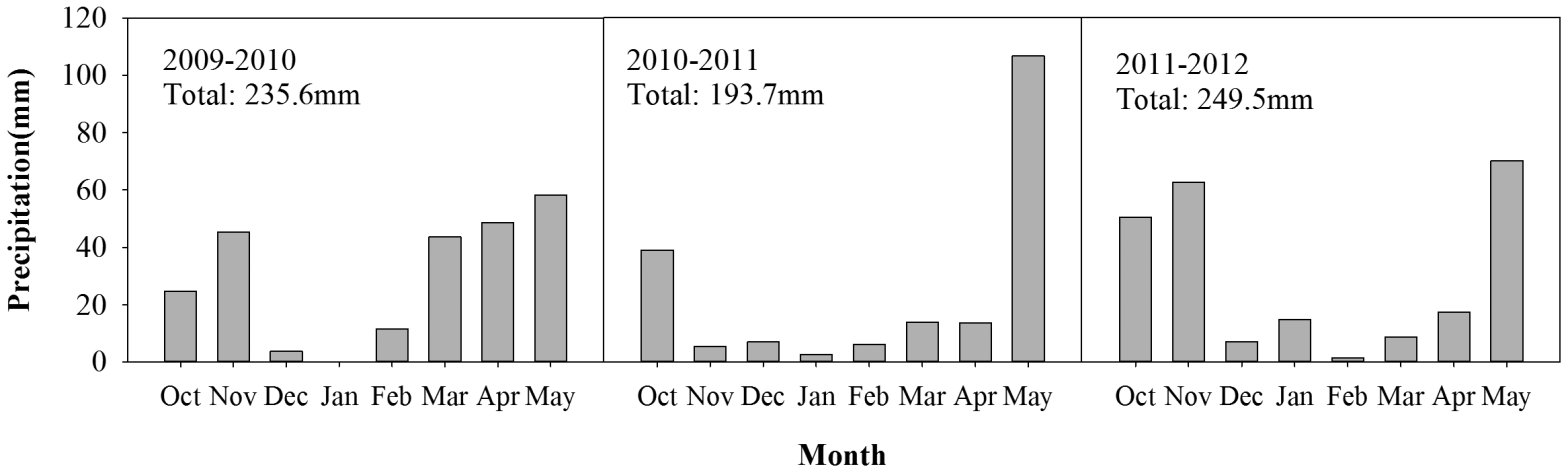
Monthly and total precipitation during the 2009–2012 wheat growing seasons. Monthly precipitation of wheat growing seasons in 2009–2010, 2010–2011, 2011–2012 in Shaanxi, Yangling.

No irrigation was used and so I = 0. Precipitation in the three growing seasons is shown in Fig. 1, and measured surface runoff was negligible during these years. Deep percolation was calculated as the difference between soil moisture content and field moisture capacity when the soil water content at this depth was more than the field water holding capacity. In the study site, deep water percolation did not occur.

WUE was defined as follows: 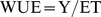



Where Y is grain yield (kg/ha).

All data concerned were analyzed by SPSS 16.0 Statistical software. ANOVA was adopted to determine whether treatments were significantly different at P<0.05. Duncan's multiple range test was used to differentiate treatment means at P<0.05.

## Results and Discussion

### Wheat yield

The yields and thousand-kernel weights of the two wheat cultivars at the different N rates in the different years are presented in Table 1. Grain yield was in the range of 2.94–7.92 t/ha for ZM and 3.39–8.19 t/ha for CH. Grain yields of the two cultivars both differed significantly between N0 and the other N rates, and increased as N rates increased, but did not differ significantly among the N-applied treatments. The lack of significant differences may due to yield in three replications being affected by other factors in field experiment. Usually the significance of increase yield is hard to attain in agricultural research, Morell et al. [36] reported that grain yield mostly 1000 kg/ha higher than control, but still have no statistical difference. The yields of wheat slightly decreased at N360, except for yield of CH in 2011. In 2010, 2011 and 2012, the wheat yields of ZM increased by up to 61.1, 118.0 and 34.7%, respectively, in the N-applied treatments compared to treatment without N fertilization; and corresponding yields of CH increased by up to 58.4, 100.8 and 51.7%. The highest yields of ZM and CH were both for treatments of N180, N270 and N270 in 2010, 2011 and 2012, respectively. Thus, N application significantly increased yields of wheat, but an excessive N rate had no positive effect on grain yield. Previous research has shown similar results with N fertilizer application significantly increasing maize and wheat yield compared to unfertilized treatments [14], [15]. Bassoa et al. [23] examined the long-term wheat response to N in rain-fed Mediterranean environments, and showed that yield response was stronger for 120 than 60 and 90 kg N/ha. Many other studies have demonstrated a parabolic relationship between N and grain yield, i.e. when N rate surpassed a certain threshold, the grain yield greatly decreased. In China, there have been many experiments on different wheat cultivars and fertilizer regimes that have shown the maximum N rate is 150–225 kg/ha. At excessive N rates, the leaf protein and chlorophyll contents decrease, and then photosynthesis also decreases [34]. Tinsina et al. [35] also showed that wheat yield was higher at 120 than 180 kg/ha in Bangladesh. Morell et al. [36] showed no additional wheat yield responses to N fertilizers at N rates >100 N kg/ha. All these studies demonstrated that N application could increase wheat yield, but excessive N had no yield benefit.

**Table 1:** Nitrogen effects on wheat yield and thousand-kernel weights of two cultivars in three years.

Varieties	Treatments	2010			2011			2012		
		Yield(kg/ha)		Thousand-kernel (g)	Yield(kg/ha)		Thousand-kernel (g)	Yield(kg/ha)		Thousand-kernel (g)
ZM	N0	4716	c	49.5 a	2974	c	47.0 a	5906	bc	49.1 a
	N90	6355	ab	42.5 b	5499	ab	42.2 ab	7272	a	42.3 bcd
	N180	7597	a	42.3 b	6355	ab	42.1 ab	7953	a	41.6 cd
	N270	7527	a	42.5 b	6482	ab	40.5 b	7923	a	41.1 d
	N360	7519	a	43.9 b	6390	ab	41.2 ab	7512	a	40.4 d
CH	N0	4162	c	46.1 ab	3391	c	42.2 ab	5407	c	46.5 ab
	N90	5862	ab	42.3 b	4886	b	41.1 ab	6926	ab	45.5 abc
	N180	6594	ab	42.3 b	5879	ab	41.9 ab	7777	a	40.4 d
	N270	6334	ab	37.9 c	6748	a	40.5 b	8199	a	40.1 d
	N360	6126	b	37.1 c	6808	a	40.3 b	8018	a	38.6 d

Values are means of three replicates for each treatment. Different letters indicate statistical significance at P<0.05 within the same column.

At the same N rates, the yields of the two cultivars did not differ significantly. In 2010, the rainfall, which was evenly distributed through the growing season, provided a more favorable environment for ZM, the water-sensitive cultivar, and so its yields were higher than those of CH for all treatments. In 2011, precipitation was the lowest in the whole growing season of all years, despite 106 mm of rainfall in May when wheat filled its seeds. However, such high rainfall at seed filling was unfavorable for wheat yield. Sheng and Wang [37] found that high soil-water contents at the seed-filling stage of wheat can result in lower thousand-kernel weights and grain yields. So drought during the growing season and too much rainfall from the seed-filling to the ripening stages led to the lower yield of wheat in 2011 compared to 2010 and 2012. Before the 2011–2012 growing season, the summer of 2011 received a lots of rain, 672.7 mm during June–September, larger than 421.6 mm in 2009 and 436.7 mm in 2010 summer. This caused total water consumption in 2011–2012 to be higher than previously and so the yields of wheat were the highest among the three years, although precipitation did not differ greatly from the growing season of 2009–2010. Shangguan et al. [38] reported that the fallow efficiencies, expressed as the ratio of soil water accumulation to precipitation received during the period of fallow, were important for yield in the next growing season. The importance of soil-water storage during the fallow period for increasing grain yields of post-fallow crops are supported by many studies on dryland including the Southern Great Plains in the USA [39]–[41] and the Loess Plateau [38]. In 2011 and 2012, the yield of ZM was higher than that of CH at N0, N90 and N180, and drought-tolerant CH showed higher yield only at the higher N rate in dry years. This is because CH was developed in recent years and prefers high fertilizer levels – consequently its cultivation has greatly expanded in northwest areas. CH is sensitive to N, and high rates of N result in higher yields; however, in contrast ZM is a poorly drought-tolerant cultivar but can produce higher yield at lower N rates. These cultivar characteristics have been demonstrated by many physiological indices in our previous studies [42]. Overall, the water consumption characteristics differed between the wheat varieties, leading to the different production performance. N180 resulted in higher yields of ZM in 2010 when rainfall was evenly distributed over the growing season. However, when rainfall was unevenly distributed or there was a lack of rainfall in the growing season, appropriate increases in the amount of N for CH could result in higher wheat yield to avoid the risk of reduced production.

The thousand-kernel weights of wheat decreased with increased N rates, consistent with many other research results [34]. There were two reasons for this: first, N can increase numbers of wheat tillers and panicles as well as flag leaf photosynthetic rates, but large and thick leaves would shade one another, affecting starch assimilation and transportation to kernels and resulting in lower thousand-kernel weights; secondly, N application could delay the flowering stage of wheat, thereby shortening the grain-filling stage and leading to lower thousand-kernel weights.

### Water consumption characteristics

#### Total ET

In dryland farming, ET is supplied partly from precipitation in the growing season and partly from soil-water storage before planting. However, the relative contribution between precipitation and crop-consumed soil water to ET differs significantly among crops.

The total ET, ΔS or rainfall and its ratio to total ET at the different N rates are shown in Table 2. Total ET behaved differently between years and cultivars. Total ET was in the range of 298.40–442.46 mm for ZM and 361.57–469.35 mm for CH. Total ET was significantly higher in the N-applied than non-N treatments, except for treatment N90. Total ET were highest in 2011–2012 of CH and ZM. In the N-applied treatments, the ET of ZM increased by up to 18.4, 15.8 and 22.1% in 2009–2010, 2010–2011 and 2011–2012, respectively, and correspondingly for CH by 28.0, 14.1 and 23.1%. The ET slightly decreased for N360, indicating that ET could not increase further if too much N was applied. Zhou et al. [15] showed that N fertilizer application decreased water storage in 0–200 cm of soil and particularly so after wheat harvesting. Hunsaker et al. [43] showed that wheat ET was significantly higher than in low N treatments. One explanation for N increasing ET of wheat is that N promotes wheat to grow more and produce longer roots, enabling more soil water to be absorbed; another explanation is that N fertilization increases the leaf area index and transpiration rates of wheat [44]. However, too much N makes soil environments stressful by increasing N concentration in soil solution, thus preventing roots from absorbing water. The total ET in this study was considerably lower than that reported for the southern high plains of the USA [45], [46] and the North China Plain [47], but was close to that for the Loess Plateau [48]. These differences are likely due to different climatic conditions, like temperature and precipitation and also attributed to different field management.

**Table 2:** Water consumption from soil (ΔS) or precipitation and its ratio to total evapotranspiration (ET) and WUE.

Year	Varieties	Treatments	Evapotranspiration (ET)(mm)		Soil water consumption (△S)		Precipitation		WUE (kg/m^3^)	
					Amount	Ratio	Amount	Ratio		
					(mm)	(%)	(mm)	(%)		
2009–2010	ZM	N0	325.72	e	90.12	27. 7	235.6	72.3	1.45	de
		N90	348.58	de	112.98	32.4		67.6	1.83	abcd
		N180	361.57	bcd	125.97	34.8		65.2	2.09	a
		N270	385.25	abc	149.65	38.9		61.2	1.96	ab
		N360	385.59	abc	149.99	38.9		61.1	1.97	ab
	CH	N0	321.39	e	85.79	26.7		73.3	1.29	e
		N90	352.69	cde	117.09	33.2		66.8	1.66	bcde
		N180	373.65	bcd	138.05	36.9		63.1	1.76	abc
		N270	411.98	a	176.38	42.8		57.2	1.48	cde
		N360	395.49	ab	159.89	40.4		59.6	1.52	cde
										
2010–2011	ZM	N0	298.40	d	104.70	35.1	193.7	64.9	1.00	d
		N90	323.28	bc	129.58	40.1		59.9	1.70	ab
		N180	344.98	ab	151.28	43.8		56.2	1.84	ab
		N270	345.60	ab	151.90	44.0		56.1	1.88	a
		N360	337.41	ab	143.71	42.6		57.4	1.89	a
	CH	N0	308.48	cd	114.78	37.2		62.8	1.10	cd
		N90	344.01	ab	150.31	43.7		56.3	1.42	bc
		N180	351.92	a	158.22	45.0		55.0	1.67	ab
		N270	351.75	a	158.05	44.9		55.0	1.92	a
		N360	344.63	ab	150.93	43.8		56.2	1.84	ab
										
2011–2012	ZM	N0	373.92	c	124.42	33.3	249.5	66.7	1.58	a
		N90	412.28	bc	162.78	39.5		60.5	1.77	a
		N180	456.45	a	206.95	45.3		54.7	1.75	a
		N270	442.06	ab	192.56	43.6		56.4	1.80	a
		N360	440.44	ab	190.94	43.3		56.6	1.71	a
	CH	N0	381.35	c	131.85	34.6		65.4	1.43	a
		N90	464.53	a	215.03	46.3		53.7	1.50	a
		N180	469.35	a	219.85	46.8		53.5	1.66	a
		N270	461.05	a	211.55	45.9		54.1	1.79	a
		N360	441.13	ab	191.63	43.4		56.6	1.82	a

Values are means of three replicates for each treatment. Different letters indicate statistical significance at P<0.05 within the same column. ΔS has the same significance as total ET.

In all the experiment years, CH had higher ET than ZM but not significantly at the same N rates – probably due to different characteristics of the varieties. As a drought-tolerant cultivar with long roots, CH is capable of absorbing deep soil water, thus presenting higher ET than ZM. A deep-growing root system will favor taking up deep soil water under water-limited conditions. Research on dryland crops has shown that deep soil-water utilization is probably limited by root density [11], [48], [49]. The rainfall was less during 2010–2011 than 2009–2010 and 2011–2012 growing seasons so that the ratio of soil water consumption was higher in the former than the other two years (Table 2). As the N application rates increased, the ratios also increased, indicating that N application helped plants utilize deeper soil water.

**Figure 2:**
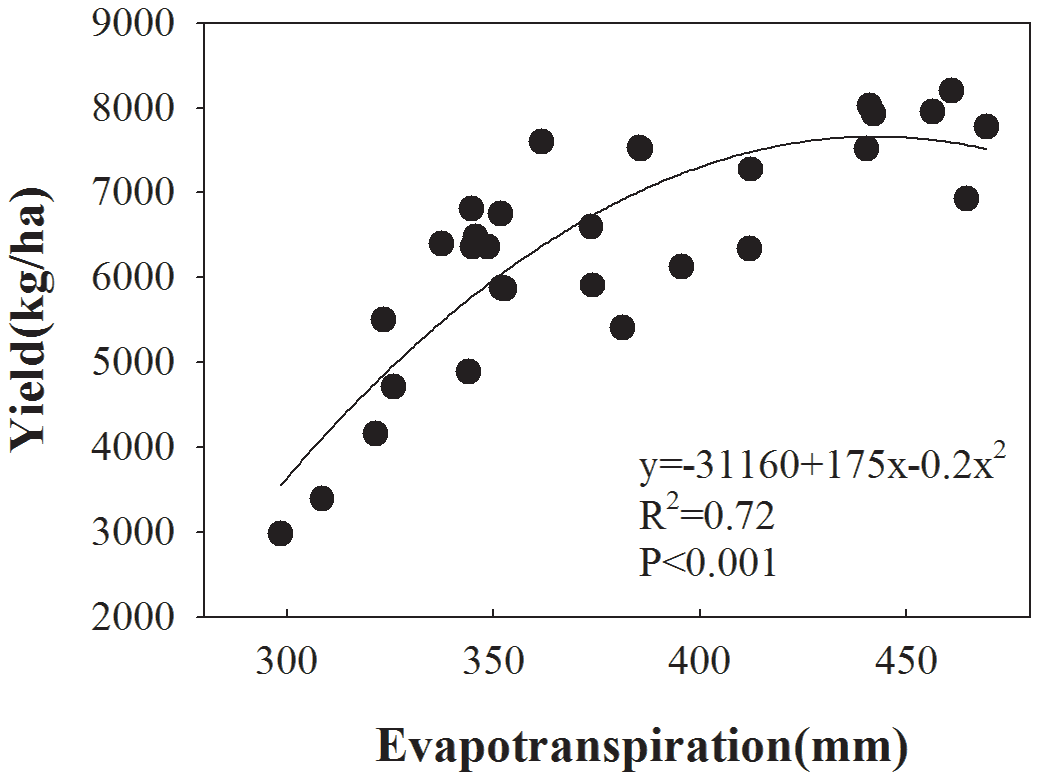
Relationship between wheat evapotranspiration (ET) (mm) and grain yield (Y) (kg/ha) for winter wheat in northwest China. The relationship between ET and yield is shown by the equation.

The relationship between grain yields and seasonal ET was best described by a quadratic function obtained by regression analysis (Y = −31160+175x−0.2x^2^; Fig. 2). Grain yield did not increase when ET exceeded a certain critical value, e.g. about 430 mm in the present study. Grain yield required a minimum ET of 244 mm for winter wheat (Fig. 2). This minimum ET value is higher than the 84 mm for wheat in the North China Plain [47] and 156 mm in the Mediterranean region [12], as well as higher than the 206 mm of dryland and irrigated wheat reported by Musick et al. [41] in US southern plains. These differences are likely due to such different climates and crop management. This result may indicate that the crop yield in this area will more relies on precipitation and soil water storage.

#### Water consumptions in the different soil layers

**Figure 3:**
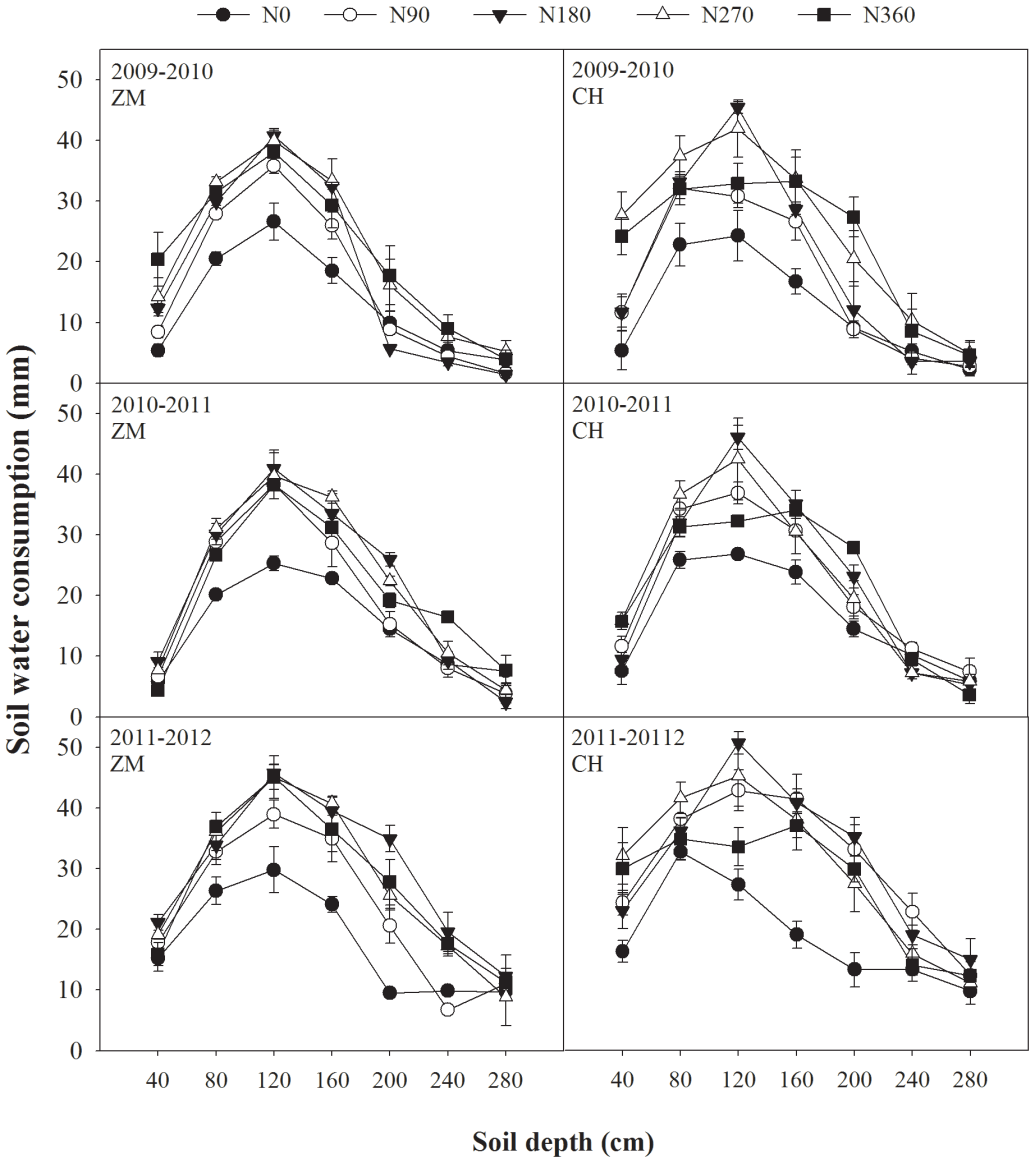
Wheat evapotranspiration (ET) (mm) of two cultivars in different soil layers and different nitrogen (N) treatments in three years. Water ET trends as soil depth increased with influence of N fertilizer for two cultivars in three years. Standard error bars are also shown.

Water consumption in the different layers of the soil profile in 40-cm increments is plotted with depth in Fig 3. N applications had a significant effect on ΔS, as well as on ET (Table 2), since N application increased water consumption in the different layers above 200 cm, except during the 2011–2012 growing season for both cultivars. In 2011–2012, at 200 cm soil depth, the N treatments still had higher soil water consumption than N0 treatment, likely due to the large amount of rainfall in this year. A similar result was found by Zhou et al. [15], with N fertilizer application decreasing water storage at soil depths of <200 cm after wheat harvesting. N application increased water consumption in the different soil layers; however, in the same soil layers, water consumption did not differ significantly among the different N rates except for some layers of CH.

Soil water consumption clearly changed with the different N rates (Fig. 3). The trends of water consumption were similar in all treatments as soil layers became deeper. N application increased water consumption in all soil layers. Generally, water consumption of CH was higher than that of ZM, which was true of the total soil water consumption in all layers (Table 2). Water was mainly consumed in layers of 40–160 cm deep, with the highest water consumption for 100–140 cm. Water was stably absorbed for soil layers <120 cm in the N0 treatment, and <160 cm in the N-applied treatments, shown by ΔS of N treatments at 160 cm being higher than for the non-N treatment at 120 cm. This was probably because N application could promote roots to grow longer and stronger, and which were able to absorb deeper soil water. In addition, the N-applied treatments had greater effects on CH than ZM (Fig. 3), showing that CH was sensitive to low N.

### WUE

WUE was in the range of 1–2.09 kg/m^3^ for ZM and 1.1–1.92 kg/m^3^ in the three years (Table 2). As the N rates increased the WUE increased, and at N360 the WUE increased slightly but not significantly. Zhou et al. [15] reported that grain yields and WUE did not significantly differ between N rates of 120 and 240 kg/ha. The above indicated that excessive N application had no favorable effect on WUE. In 2009–2010 and 2010–2011, the N-applied treatments significantly improved WUE. However, in 2011–2012, WUE did not significantly differ between the N-applied and non-N treatments. Compared to 2009–2010 and 2010–2011, the WUE of the non-N treatment was higher in 2011–2012. This may be due to the higher soil water content before sowing and the higher rainfall in the growing season in 2012 that increased the grain yield in the non-N treatment. Consequently higher WUE in non-N treatment reduced the difference between non-N and N-applied treatments.

The WUEs obtained in the present study were higher than those of irrigated winter wheat (0.40–0.88 kg/m^3^) [45], [46] and of irrigated wheat in the US southern plains (0.82 kg/m^3^) [41], as well as higher than these of irrigated wheat in the Loess Plateau (0.73–0.93 kg/m^3^) [14], demonstrating that in dryland farming systems N fertilizer can be a useful way to increase WUE. However, the results of the present study were similar to those of winter wheat in the North China Plain (0.84–1.39 kg/m^3^) [12] and of N-fertilized winter wheat in Yangling, Shaanxi (0.8–1.5 kg/m^3^) [15]. These differences are caused by different climate or water, fertilizer and crop management. N fertilizer application significantly increases the yield and WUE of both wheat and maize, indicating that N fertilizer application is an effective way to increase grain yield in the study region. Deng et al. [50] reviewed four published research reports and found that N fertilizers increased WUE of wheat and potato by an average of 20% in north central and northwest China.

**Figure 4:**
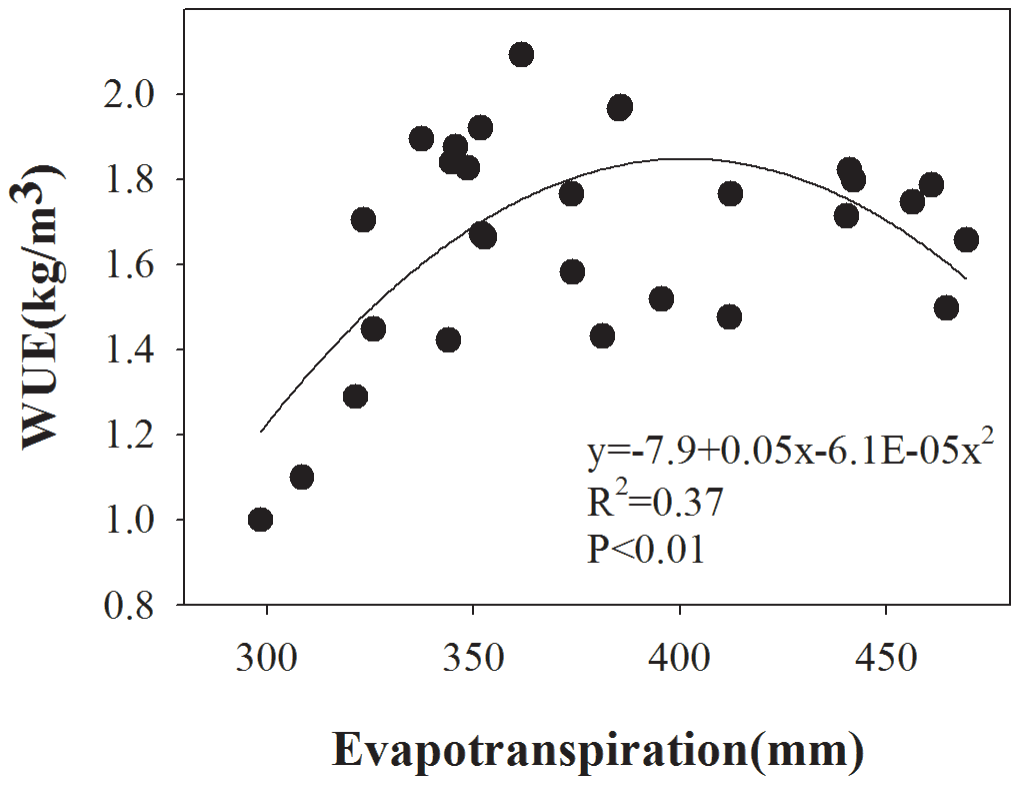
Relationship between evapotranspiration (ET) (mm) and WUE (Y) for winter wheat in northwest China. The relation between ET and WUE is shown by the equation.

**Figure 5:**
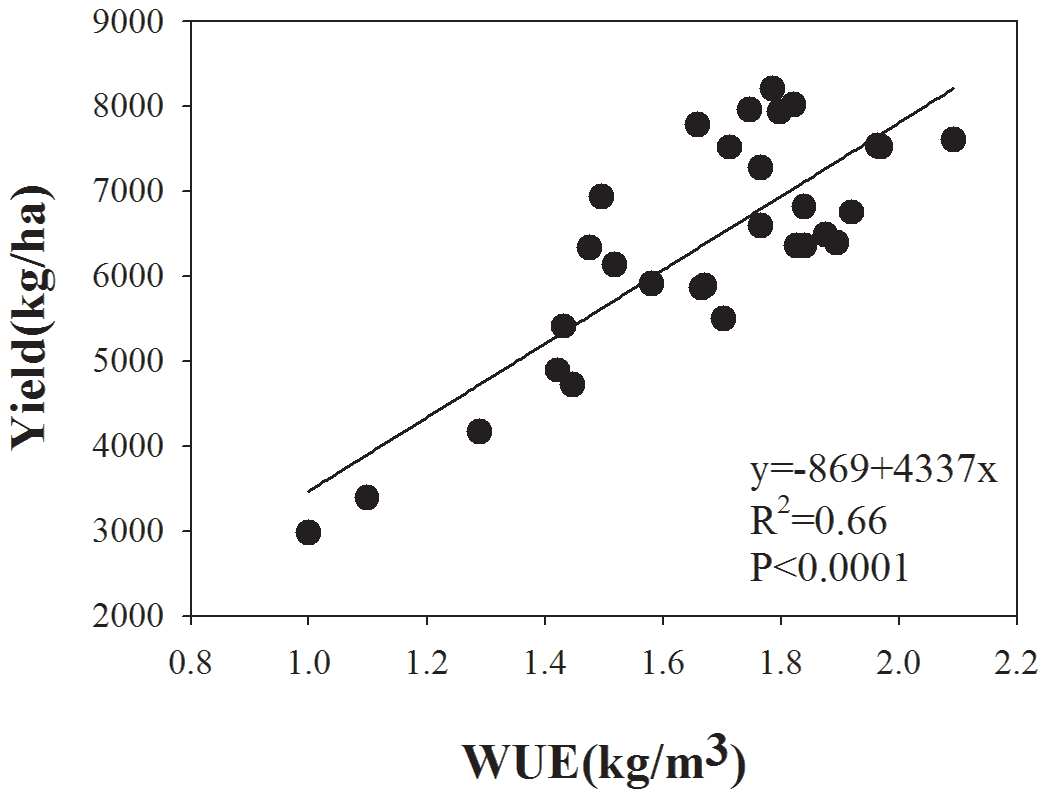
Relationship between WUE and grain yield (Y) for winter wheat in northwest China. The relation between WUE and yield could be deduced from the liner equation.

Regression analysis produced a quadratic relationship between ET and WUE (Fig. 4) and correlations were calculated between WUE and wheat yields (Fig. 5). The yields increased linearly with WUE: WUE reached a maximum value at the ET of 401 mm and then decreased (Fig. 4). However, the maximum WUE did not correspond to the maximum grain yield in the study – the higher WUE means that the crop can gain high yield using less water. This is an important method to obtain a balance between higher yields and lower water supplies of wheat in arid and semi-arid regions by increasing its WUE. In the present study, although there were significant differences between N treatments, both cultivars had relatively higher WUEs at the N rate of 270 kg/ha in a dry year.

## Conclusions

N fertilization affected the grain yields, thousand-kernel weights, ET and WUE of the two different water-sensitive wheat cultivars, ZM and CH. The most common pattern of farming in northwest China was used in the present study, with long-term different rates of N fertilization and no irrigation during the wheat growing season. We concluded that (1) in this dryland farming system, increased N fertilization resulted in higher wheat yields in a situation of low precipitation; the drought-tolerant CH showed a yield advantage in a drought environment with high N fertilizer rates; (2) N application affected water consumption in the different soil layers, and promoted absorption and utilization of water from deeper soil layers; and (3) comprehensive consideration of yield and WUE of wheat indicated that the N rate of 270 kg/ha for CH was better to avoid the risk of reduced production due to lack of precipitation; however, under conditions of better soil moisture, the N rate of 180 kg/ha was more economic.
